# Impact of routine bedside infectious disease consultation on clinical management and outcome of *Staphylococcus aureus* bacteraemia in adults

**DOI:** 10.1016/j.cmi.2015.05.026

**Published:** 2015-08

**Authors:** R.B. Saunderson, T. Gouliouris, E.K. Nickerson, E.J.P. Cartwright, A. Kidney, S.H. Aliyu, N.M. Brown, D. Limmathurotsakul, S.J. Peacock, M.E. Török

**Affiliations:** 1) Department of Dermatology, Royal North Shore Hospital, St Leonards, Australia; 2) Department of Medicine, University of Cambridge, UK; 3) Clinical Microbiology and Public Health Laboratory, Public Health England, UK; 4) Department of Infectious Diseases, Cambridge University Hospitals NHS Foundation Trust, Cambridge, UK; 5) Mahidol-Oxford Tropical Medicine Research Unit, Mahidol University, Bangkok, Thailand

**Keywords:** Bacteraemia, infectious disease consultation, outcome, *Staphylococcus aureus*, treatment

## Abstract

*Staphylococcus aureus* bacteraemia (SAB) is a common, serious infection that is associated with high rates of morbidity and mortality. Evidence suggests that infectious disease consultation (IDC) improves clinical management in patients with SAB. We examined whether the introduction of a routine bedside IDC service for adults with SAB improved clinical management and outcomes compared to telephone consultation. We conducted an observational cohort study of 571 adults with SAB at a teaching hospital in the United Kingdom between July 2006 and December 2012. A telephone consultation was provided on the day of positive blood culture in all cases, but an additional bedside IDC was provided after November 2009 (routine IDC group). Compared to patients in the pre-IDC group, those in the routine IDC group were more likely to have a removable focus of infection identified, echocardiography performed and follow-up blood cultures performed. They also received longer courses of antimicrobial therapy, were more likely to receive combination antimicrobial therapy and were more likely to have SAB recorded in the hospital discharge summary. There was a trend towards lower mortality at 30 days in the routine IDC group compared to the pre-IDC group (12% vs. 22%, p 0.07). Our findings suggest that routine bedside IDC should become the standard of care for adults with SAB.

## Introduction

*Staphylococcus aureus* bacteraemia (SAB) is a common, serious infection with mortality rates of up to 40% [1–3]. Several retrospective studies have shown improvements in the clinical management and outcome of patients with bacteraemia who receive infectious diseases specialist advice. Infectious disease specialist advice for patients with bacteraemia increases the prescription of correct empirical therapy [4–7] and results in a narrowing of the spectrum of antimicrobial therapy [5]. In patients with SAB, infectious disease consultation (IDC) leads to improvements in clinical management [8–11], is associated with lower rates of recurrent disease [8,9], is associated with reductions in mortality [11,12] and is cost effective [13,14]. One study has also compared telephone consultation with bedside IDC in the management of SAB [15]. A recent review article has recommended that all adult patients with SAB should undergo echocardiography [16].

In the United Kingdom, the management of SAB varies widely between different hospitals. When blood cultures become positive, clinical microbiologists usually provide telephone advice to the primary clinical care team. Bedside clinical review is not routine clinical practice, and often only occurs in centres with infectious diseases services. The aim of this study was to evaluate whether the introduction of routine bedside IDC service for adults with SAB resulted in improvements in management and outcomes compared to telephone consultation.

## Patients and methods

### Study setting and participants

An observational cohort study of adults with SAB was conducted at the Cambridge University Hospitals National Health Service Foundation Trust (CUHNHFT), a tertiary-care referral centre. All first episodes of SAB in patients older than 18 years admitted between July 2006 and December 2012 were eligible for inclusion in the study. The exclusion criteria included death before blood cultures becoming positive, transfer to another hospital before the blood cultures became positive, polymicrobial cultures or positive cultures that were deemed to be contaminated.

### Study procedures

On the day the blood culture became positive, the clinical microbiologist provided telephone advice to the primary clinical team. Before November 2009, patients were not routinely reviewed by the infectious diseases service; an IDC only occurred if requested by the primary clinical team, and this usually consisted of a single bedside review. For patients admitted between July 2006 and November 2009 (the pre-IDC group), data were collected retrospectively from the medical records and a national register of deaths. In November 2009, a routine bedside IDC service was introduced for all patients with SAB. In addition to the telephone advice provided by the microbiology team, patients received an initial bedside clinical review, followed by weekly clinical review by the IDC team until the time of hospital discharge. Written recommendations for investigation and management were recorded in the medical records. Data in this group were collected prospectively.

### Study definitions and outcome measures

Previously defined criteria were used to determine if the source of the bacteraemia was due to soft tissue infection [8], intravascular (iv) catheter infection [17], native and prosthetic valve infective endocarditis [18] or bone and joint infections (US Centers for Disease Control and Prevention, http://www.cdc.gov/nhsn/PDFs/pscManual/17pscNosInfDef_current.pdf). For all other foci of infection, clinical and/or radiologic and/or microbiologic evidence was used to determine the source of infection. Community-acquired, hospital-acquired and healthcare-associated infections were defined according to previously published criteria [19]. Uncomplicated SAB was defined as negative blood culture results 2 to 4 days after the initial positive blood culture finding and/or defervescence at 72 hours, and no evidence of endocarditis, metastatic disease or deep focus of infection [20]. Complicated SAB cases did not meet the criteria for uncomplicated infection, had an unknown focus or died within 72 hours. Recurrence was defined as blood cultures positive for *S. aureus* 14 days after the initial positive result. Metastatic infection was defined as a secondary site of infection distant from the primary site of infection.

Appropriate antimicrobial therapy was defined as therapy to which the isolate was determined susceptible by antimicrobial susceptibility testing. β-Lactam therapy was recommended for methicillin-susceptible SAB, and vancomycin was recommended for methicillin-resistant SAB unless the patient had a contraindication to this. The duration of therapy was the length of time in days that the patient received antibiotics to which the isolate was susceptible, and was measured from the time when the blood culture result became available. Patients who died while receiving antibiotic therapy were excluded from the analyses of antibiotic duration. Combination antibiotic therapy was prescribed for certain infections including infective endocarditis, and bone and joint infections [21–23].

The presence of comorbidities was assessed using the Charlson comorbidity index (CCI) and were dichotomized into scores of <3 or ≥3 [24,25]. Immunosuppressive therapy included corticosteroid therapy administered at a minimum dose equivalent to 5 mg prednisone for a minimum period of 2 weeks, neutropenia from any cause, treatment with antineoplastic therapy or a biologic agent or patients undergoing plasmapheresis. Chronic skin conditions were defined as those conditions that predisposed the subject to colonization with *S. aureus.*

Evidence from previous studies was used to determine quality indicators for the investigation and management of SAB. These included: (a) identification and removal of a removable focus of infection [8,26]; (b) performing repeat blood cultures at 48 to 96 hours [27]; (c) performing an echocardiogram [27,28]; (d) use of β-lactam therapy for methicillin-susceptible SAB [29,30]; (e) treatment of uncomplicated infection with 14 days of iv antibiotics [26]; and (f) treatment of complicated infection with a minimum of 28 days of iv antibiotics [22,26]. We also noted whether the SAB was recorded in the hospital discharge summary.

The following outcome measures were assessed: (a) defervescence within 72 hours; (b) duration of hospital stay; (c) death at 30 and 90 days after first positive blood culture; and [4] recurrent SAB at 30 and 90 days after first positive blood culture. Death was attributed to SAB if there were symptoms and signs consistent with SAB at the time of death, if there was a persistent focus of *S. aureus* infection at the time of death and/or if the blood culture was positive at the time of death.

### Statistical methods

Data were analysed by Stata, version 12 (StataCorp, USA). Continuous variables were reported as median and interquartile range, and categorical variables reported as number and percentage. The Fisher exact test was used to compare categorical variables and the Mann-Whitney test to compare continuous variables. Time-to-event outcomes were analysed by Kaplan-Meier survival analysis and the Cox proportional hazard model. A multivariable Cox proportional hazard model, adjusted for age, sex, hospital-acquired infection, methicillin-resistant *S. aureus* (MRSA) and CCI, was used to compare the 30- and 90-day mortality between the two groups.

### Ethics statement

The study was approved by the University of Cambridge Human Biology Research Ethics Committee (reference HBREC.2013.05) and by the CUHNHFT Research and Development Department (reference A092869).

## Results

### Baseline characteristics

**Fig. 1:**
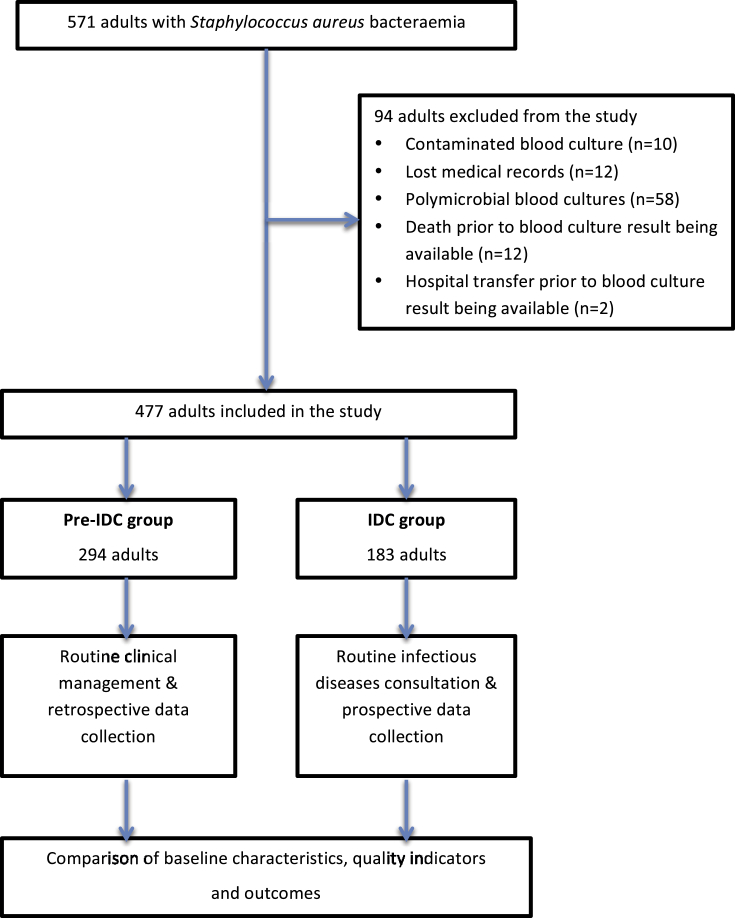
Flow chart of study participants.

A total of 571 adults with SAB between 16 July 2006 and 31 December 2012 were eligible for inclusion in the study. Ninety-four subjects were excluded for various reasons, leaving 477 in the final analysis (Fig. 1). There were 294 adults in the pre-IDC group (60 of whom received a bedside IDC requested by the primary clinical team) and 183 patients in the routine IDC group (178 of whom received an IDC). Of the five patients who did not receive an IDC, three patients died before IDC, one patient was transferred to another hospital before IDC and one patient was discharged from hospital after 1 day and completed treatment in the community. The baseline characteristics of the two groups were similar for most criteria (Table 1). The proportion of patients with prosthetic material was higher in the routine IDC group compared to the pre-IDC group (55% vs. 44%, p 0.02). The proportion of patients who had a CCI score of ≥3 was higher in the pre-IDC group compared to the routine IDC group (48% vs. 38%, p 0.03).

### Clinical features of SAB

Overall, 46% of SAB cases were hospital acquired, 27% were healthcare associated and 28% were community acquired (Table 2). The frequency of hospital-acquired SAB was higher in the pre-IDC group (52% vs. 36%, p < 0.001), as was the frequency of MRSA compared to methicillin-susceptible *S. aureus* bacteraemia (34% vs. 14%, p < 0.001). The most common foci of infection were skin and soft tissue infections (31%), central venous catheters (23%) or unknown foci (14%) (Table 2). The proportion of patients with an unknown focus was higher in the pre-IDC group (16% vs. 9%, p 0.04). Certain foci of infection (e.g. thrombophlebitis, implanted vascular device, joint infection, nonvertebral osteomyelitis, skin and soft tissue infections) were more frequently identified in the routine IDC group compared to the pre-IDC group (Table 2). The frequency of uncomplicated infection was similar in each group (24% vs. 20%, p 0.31).

### Quality indicators for management of SAB

The median time to receive an IDC was shorter in the routine IDC group (2 vs. 3 days, p < 0.001) (Table 3). A repeat blood culture was performed more frequently in the routine IDC group (93% vs. 65%, p < 0.001). A removable focus of infection was more frequently identified in the routine IDC group (49% vs. 40%, p 0.05), although there was no significant difference in the proportion of patients who had the focus removed. Echocardiography was performed more frequently in the routine IDC group (91% vs. 38%, p < 0.001). Combination antibiotic therapy was prescribed more frequently in the routine IDC group (24% vs. 16%, p 0.04). There was no difference in the use of β-lactam therapy for methicillin-susceptible SAB between the two groups.

The median duration of antibiotic therapy was 19.5 days for iv therapy and 28 days for the total duration of therapy (iv and oral). The duration of therapy was longer in the routine IDC group, both for iv antibiotic therapy (29 vs. 15 days, p < 0.001) and for total duration of therapy (31 vs. 21 days, p < 0.001). The duration of treatment for both uncomplicated and complicated infection was longer in the routine IDC group. For uncomplicated infections, the duration of iv antibiotic therapy (21 vs. 14.5 days, p < 0.001) and total antibiotic therapy (22 vs. 18 days, p 0.07) was longer in the routine IDC group compared to the pre-IDC group. For complicated infections, the duration of iv antibiotic therapy (29 vs. 16 days, p < 0.001) and total antibiotic therapy (34 vs. 24 days, p < 0.001) was also longer in the routine IDC group than the pre-IDC group. SAB was more frequently recorded in the discharge summary in the routine IDC group (75% vs. 41%, p < 0.001).

### Outcome from SAB

**Fig. 2:**
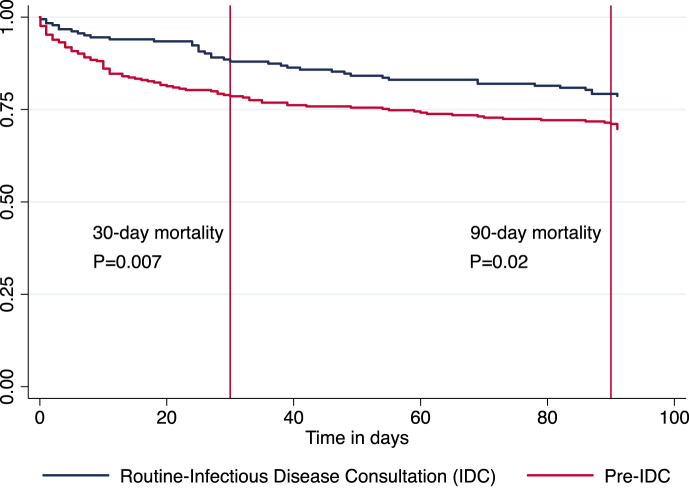
Kaplan-Meier survival estimate for adults with SAB.

Length of hospital stay and defervescence within 72 hours were similar between groups (Table 3). In univariable analyses, mortality at 30 and 90 days was lower in the routine IDC group (Table 4 and Fig. 2). In a multivariable Cox proportional hazard model adjusted for sex, age, hospital-acquired infection, MRSA infection and CCI, we found that there was a trend towards lower mortality at 30 days in the routine IDC group (12% vs. 22%, hazard ratio 0.62, 95% confidence interval 0.37–1.03, p 0.07) and at 90 days (21% vs. 30%, hazard ratio 0.85, 95%, confidence interval 0.57–1.28, p 0.44). Older age and higher CCI scores were associated with a higher mortality at 30 days. Increased age, MRSA infection and CCI scores were associated with 90-day mortality. Death was attributed to SAB in a higher proportion in the pre-IDC group (56% vs. 23%, p < 0.001). There was no significant difference in recurrent SAB between the two groups.

## Discussion

Our study findings support the use of routine bedside IDC for the management of adults with SAB and confirmed that a bedside IDC is superior to telephone consultation [15]. Patients in the routine IDC group were more likely to have a removable focus of infection identified, echocardiography performed and follow-up blood cultures performed. They received longer courses of antimicrobial therapy, were more likely to be receive combination antimicrobial therapy and were more likely to have SAB recorded in the hospital discharge summary. In contrast to previous studies, we provided an initial clinical review followed by weekly inpatient follow-up until the time of hospital discharge. This enabled us to provide ongoing clinical advice and to monitor compliance with our recommendations.

Patients in the routine IDC group received longer courses of antibiotic therapy for both complicated and uncomplicated infections. Patients in the routine IDC group received 4 to 6 weeks of iv antimicrobial therapy for complicated infection, in accordance with current treatment recommendations. This was an improvement compared to the pre-IDC group. In uncomplicated infection, however, there was a tendency to treat patients for longer than 14 days of iv therapy. This was an unexpected finding and suggests that although these patients fulfilled the study-defined criteria for uncomplicated infection, a longer treatment course was administered. Possible explanations include the attending infectious disease specialist recommending a longer course of therapy on the basis of subsequent data (e.g. evolving clinical features, failure to respond to treatment) or the primary clinical care team deferring the decision to stop antimicrobial therapy until the patient received his or her weekly review by the IDC team.

The frequency of hospital-acquired bacteraemia was lower in the routine IDC group. This may be explained by a decline in MRSA bacteraemia after the introduction of infection control measures (MRSA screening and decolonization of inpatients), as well as the introduction of a vascular catheter insertion service at our hospital during the study period.

Inpatient mortality and death at 30 and 90 days were higher in the pre-IDC group. These findings may have been related to improvements in clinical management or changes in risk factors over time. A higher proportion of patients in the pre-IDC group had MRSA bacteraemia and a CCI score of ≥3, suggesting the pre-IDC group was a more unwell cohort. Nonetheless, there was a trend towards reduced mortality in multivariable analysis in the routine IDC group. This may have been related to a decrease in the proportion of patients with an unknown focus, which is known to be associated with increased mortality. Fewer deaths were attributed to SAB in the routine group, which may reflect improved clinical management during this period.

We acknowledge several limitations to our study. We aimed to minimize selection bias by including all adults with SAB and collecting data using a standard case record form. However, data from the pre-IDC group were collected retrospectively from existing documentation, which may not have been as comprehensive as that recorded prospectively by the IDC team. The time period of the study (2006–2009 for the pre-IDC group and 2009–2012 for the routine IDC group) may have been associated with confounding factors such as introduction of clinical services (e.g. vascular catheter insertion service). Nevertheless, our findings suggest that routine regular bedside IDC is superior to a telephone consultation for the management of SAB in adults and should become the standard of care.

## Transparency declaration

This study was funded by grants from the United Kingdom Clinical Research Collaboration (UKCRC) Translational Research Initiative (Medical Research Council – MRC grant G1000803), the Hospital Infection Society and the National Institute of Health Research (NIHR) Cambridge Biomedical Research Centre. MET is a Clinician Scientist Fellow funded by the Academy of Medical Sciences and the Health Foundation. All authors report no conflicts of interest relevant to this article.

## Figures and Tables

**Table 1:** Baseline characteristics of adults with *Staphylococcus aureus* bacteraemia

Characteristic	All patients (*N* = 477)	Before IDC (*n* = 294)	Routine IDC (*n* = 183)	p
Age (years)	65.6 (50.4–76.4)	65.8 (50.5–77.4)	64.8 (50.2–75.3)	0.47
Male sex	287 (60.2)	184 (62.6)	103 (56.3)	0.18
Duration of bacteraemia symptoms before treatment				0.005
0–24 hours	220 (46.1)	152 (51.7)	68 (37.2)	
25–72 hours	80 (16.8)	38 (12.9)	42 (23.0)	
>73 hours	141 (29.6)	85 (28.9)	56 (30.6)	
Unknown	36 (7.6)	19 (6.5)	17 (9.3)	
Surgery in last 30 days	93 (19.5)	65 (22.1)	28 (15.3)	0.08
Comorbidity/risk factor				
Diabetes mellitus	104 (21.8)	55 (18.7)	49 (26.8)	0.04
Cardiac failure	50 (10.5)	31 (10.5)	19 (10.4)	1.00
Ischaemic heart disease	75 (15.7)	49 (16.7)	26 (14.2)	0.52
Peripheral vascular disease	36 (7.6)	22 (7.5)	14 (7.7)	1.00
Cerebrovascular disease	66 (13.8)	46 (15.7)	20 (10.9)	0.17
Chronic pulmonary disease	93 (19.5)	64 (21.8)	29 (15.9)	0.12
Liver disease	65 (13.6)	41 (14.0)	24 (13.1)	0.89
Malignancy	110 (23.1)	72 (24.5)	38 (20.8)	0.37
Organ transplant	22 (4.6)	9 (3.1)	13 (7.1)	0.05
HIV infection	3 (0.63)	2 (0.7)	1 (0.6)	1.00
Intravenous drug abuse	26 (5.5)	12 (4.1)	14 (7.7)	0.10
Immunosuppression	115 (24.1)	62 (21.1)	53 (29.0)	0.06
Moderate to severe renal disease	96 (20.1)	63 (21.4)	33 (18.0)	0.48
Chronic skin condition	86 (18.0)	41 (14.0)	45 (24.6)	0.005
Prosthetic material	229 (48.0)	129 (43.9)	100 (54.6)	0.02
Charlson comorbidity index	2 (1–4)	2 (1–5)	2 (1–4)	0.02
Score ≥3	210 (44.0)	141 (48.0)	69 (37.7)	0.03

Data are presented as median (interquartile range) or *n* (%). IDC, infectious disease consultation.

**Table 2:** Clinical features of infection in adults with *Staphylococcus aureus* bacteraemia

Characteristic	All patients (*N* = 477), *n* (%)	Before IDC (*n* = 294), *n* (%)	Routine IDC (*n* = 183), *n* (%)	p
Acquisition of infection				
Community acquired	131 (27.5)	67 (22.8)	64 (35.0)	0.004
Healthcare associated	129 (27.0)	75 (25.5)	54 (29.5)	0.34
Hospital acquired	217 (45.5)	152 (51.7)	65 (35.5)	<0.001
MRSA	127 (26.6)	101 (34.4)	26 (14.2)	<0.001
Focus of infection				
Unknown	65 (13.6)	48 (16.3)	17 (9.3)	0.04
Central venous catheter	110 (23.1)	70 (23.8)	40 (21.9)	0.66
Peripheral venous catheter	39 (8.2)	24 (8.2)	15 (8.2)	1.0
Thrombophlebitis	35 (7.3)	12 (4.1)	23 (12.6)	0.001
Implanted vascular device	4 (0.84)	0 (0)	4 (2.2)	0.02
Infective endocarditis	26 (5.5)	12 (4.1)	14 (7.7)	0.10
Native valve	18 (3.8)	8 (2.7)	10 (5.5)	0.14
Prosthetic valve	8 (1.7)	4 (1.4)	4 (2.2)	0.5
Joint infection	45 (9.4)	21 (7.1)	24 (13.1)	0.04
Native joint	24 (5.0)	11 (3.7)	13 (7.1)	0.13
Prosthetic joint	21 (4.4)	10 (3.4)	11 (6.0)	0.25
Vertebral osteomyelitis	33 (6.9)	17 (5.8)	16 (8.7)	0.27
Epidural abscess	23 (4.8)	13 (4.4)	10 (5.5)	0.66
Osteomyelitis (nonvertebral)	24 (5.0)	10 (3.4)	14 (7.7)	0.05
Skin and soft tissue infection	147 (30.8)	81 (27.6)	66 (36.1)	0.05
Deep tissue abscess	24 (5.0)	18 (6.1)	6 (3.3)	0.20
Lung	40 (8.4)	32 (10.9)	8 (4.4)	0.02
Urogenital	22 (4.6)	15 (5.1)	7 (3.8)	0.66
Central nervous system	11 (2.3)	5 (1.7)	6 (3.3)	0.35
Mediastinitis	5 (1.1)	2 (0.68)	3 (1.6)	0.38
Other	10 (2.1)	6 (2.0)	4 (2.2)	1.0
Complicated infection	371 (77.8)	224 (76.2)	147 (80.3)	0.31
Metastatic infection				
At presentation	33 (6.9)	21 (7.1)	12 (6.6)	0.86
Ever occurred	58 (12.2)	33 (11.2)	25 (13.7)	0.47

IDC, infectious disease consultation.

**Table 3:** Quality indicators and outcomes in the management of *Staphylococcus aureus* bacteraemia

Characteristic	All patients (*N* = 477)	Before IDC (*n* = 294)	Routine IDC (*n* = 183)	p
**Quality Indicators**				
IDC				
Bedside IDC	238 (49.9)	60 (20.4)	178 (97.3)	<0.001
Telephone-only consultation	239 (50.1)	234 (79.6)	5 (2)	<0.001
Time to bedside IDC (days)	2 (1–3)	3 (1–9)	2 (1–3)	<0.001
Repeat blood culture performed	361 (75.7)	191 (65.0)	170 (92.9)	<0.001
Removable focus of infection	205 (43.0)	116 (39.5)	89 (48.6)	0.06
Focus of infection removed	190 (92.7)	108 (93.1)	82 (92.1)	0.79
Echocardiogram				
First echocardiogram performed	279 (58.5)	113 (38.4)	166 (90.7)	<0.001
TTE	273 (95.1)	114 (98.3)	159 (93.0)	0.25
TOE	14 (4.9)	2 (1.7)	12 (7.0)	<0.001
Days to echocardiogram	7 (4–10)	7 (4–12)	7 (4–9)	0.54
Subsequent echocardiogram	40 (8.3)	9 (3.1)	31 (16.9)	<0.001
TTE	24	2	22	<0.001
TOE	16	7	9	0.19
Antibiotic therapy				
Duration of iv therapy (days)^a^	19.5 (13–31)	15 (10–28)	29 (17 -42)	<0.001
Complicated infection	22 (13–35)	16 (10–29)	29 (18–45)	<0.001
Uncomplicated infection	16 (11–21)	14.5 (10–19)	21 (15–30)	<0.001
Duration total therapy (days) ^3^	28 (16–42)	21 (14 - 32)	31 (20–48)	<0.001
Complicated infection	29 (17–45)	24 (14–41)	34 (28–53)	<0.001
Uncomplicated infection	19 (15–29)	18 (14–29)	22 (16–30)	0.07
β-Lactam therapy for MSSA^b^	310/349 (71.9)	170/193 (88.1)	140/156 (89.7)	0.73
Combination antibiotic therapy	92 (19.3)	48 (16.3)	44 (24.0)	0.04
SAB recorded in discharge summary	259 (54.3)	121 (41.2)	138 (75.4)	<0.001
**Outcome Measures**				
Defervescence within 72 hours	293/442 (66.3)	185/271 (68.3)	108/171 (63.2)	0.30
Length of hospital stay (days)^c^	29 (17–52)	30 (16–51)	29 (17–53)	0.89
Mortality				
30 day	86 (18)	64 (21.9)	22 (12)	0.007
90 day	128 (26.8)	89 (30.3)	39 (21.3)	0.03
Death attributed to SAB	59/128 (46.0)	50/89 (56.2)	9/39 (23.1)	<0.001
Recurrent disease				
30 day	4 (0.84)	4 (1.4)	0 (0)	0.30
90 day	16 (3.4)	12 (4.1)	4 (2.2)	0.31

Data are presented as median (interquartile range) or *n* (%). IDC, infectious disease consultation; iv, intravenous; MSSA, methicillin-susceptible *S. aureus;* SAB, *S. aureus* bacteraemia; TOE, transoesophageal echocardiogram; TTE, transthoracic echocardiogram.

a Calculation excluded patients who died while receiving active treatment with antibiotic therapy.

b Calculation based on patients with MSSA.

c Calculation excluded patients who died in hospital.

**Table 4:** Factors associated with 30- and 90-day mortality

Factor	Univariable model		Multivariable model	
	Crude hazard ratio (95% CI)	p	Adjusted hazard ratio (95% CI)	p
30-day mortality				
IDC	0.51 (0.32–0.83)	0.007	0.62 (0.37–1.04)	0.07
Age	1.03 (1.02–1.05)	<0.001	1.03 (1.02–1.05)	<0.001
Male sex	0.90 (0.58–1.39)	0.63	1.00 (0.64–1.58)	0.97
Hospital-acquired infection	1.15 (0.76–1.76)	0.51	0.88 (0.56–1.38)	0.57
MRSA	1.98 (1.29–3.05)	0.003	1.55 (0.98–2.45)	0.06
CCI score >3	2.88 (1.83–4.52)	<0.001	2.42 (1.52–3.85)	<0.001
90-day mortality				
IDC	0.65 (0.44–0.94)	0.02	0.85 (0.57–1.28)	0.44
Age	1.04 (1.03–1.05)	<0.001	1.04 (1.02–1.05)	<0.001
Male sex	0.97 (0.68–1.38)	0.85	1.06 (0.74–1.53)	0.76
Hospital-acquired infection	1.49 (1.05–2.11)	0.03	1.20 (0.82–1.74)	0.35
MRSA	2.01 (1.41–2.87)	<0.001	1.56 (1.07–2.29)	0.02
CCI score >3	3.40 (2.33–4.96)	<0.001	2.89 (1.96–4.25)	<0.001

CCI, Charleston comorbidity index; CI, confidence interval; IDC, infectious disease consultation; MRSA, methicillin-resistant *S. aureus*.
